# Faecal microbiota and fatty acids in feline chronic enteropathy

**DOI:** 10.1186/s12917-023-03824-9

**Published:** 2023-12-20

**Authors:** Julia Miller, Paulina Żebrowska-Różańska, Aleksandra Czajkowska, Bogumiła Szponar, Aleksandra Kumala-Ćwikła, Magdalena Chmielarz, Łukasz Łaczmański

**Affiliations:** 1 Department of Immunology, Pathophysiology and Veterinary Preventive Medicine, Wroclaw University of Environmental and Life Sciences, Norwida 31, Wroclaw, 50-375 Poland; 2 Ludwik Hirszfeld Institute of Immunology and Experimental Therapy, Polish Academy of Sciences, Wroclaw, Poland; 3 Department of Basic Sciences, Faculty of Health Sciences, Wroclaw Medical University, Wroclaw, Poland; 4 Veterinary clinic “Z pazurem”, Krakow, Poland

## Abstract

**Background:**

Feline chronic enteropathy is a set of disorders defined as the presence of clinical signs of gastrointestinal disease for at least three weeks. The most common final diagnoses are inflammatory bowel disease and alimentary small cell lymphoma. The etiopathogenesis of these diseases is incompletely understood; however, it is hypothesised that they involve a combination of factors, including altered composition and/or functionality of the intestinal microbiome. An important factor in the interplay of the microbiome and host is the production of short- and branched-chain fatty acids.

The aim of this study was to evaluate the possible differences in faecal microbiota diversity, composition and fatty acid production between cats suffering from chronic enteropathy and healthy cats. Sixteen cats suffering from chronic enteropathy and fourteen healthy control cats were enrolled in the study. The microbiota compositions of faecal samples were analysed by using next-generation amplicon sequencing of the V3V4 fragment of the 16S rRNA gene. Fatty acids were evaluated by high-performance liquid chromatography.

**Results:**

Both the alpha and beta diversities were significantly lower in samples obtained from cats with chronic enteropathy. The relative abundance of the phylum Proteobacteria, orders Lactobacillales and Enterobacterales, family *Enteriobacteriaceae* and genus *Escherichia Shigella* were higher in diseased cats, whereas the abundance of the phylum Bacteroidota and order Peptococcales were higher in control cats. The faecal concentrations of short-chain fatty acids were higher in cats with chronic enteropathy, with lower propionate proportions and higher butyrate proportions.

**Conclusion:**

The study revealed alterations in microbiota compositions and short-chain fatty acid concentration in cats suffering from chronic enteropathy, which is an important finding both for research on the pathogenesis of the disease and for potential therapeutic interventions in the form of faecal microbiota transplantation and/or probiotic supplementation.

**Supplementary Information:**

The online version contains supplementary material available at 10.1186/s12917-023-03824-9.

## Background

Feline chronic enteropathy (CE) is a set of disorders defined as the presence of clinical signs of gastrointestinal (GI) disease for at least three weeks with no evidence of extra-intestinal disease causing secondary GI signs [1]. The most common final diagnoses in CE (based on histopathological examinations) are inflammatory bowel disease (IBD) and alimentary small cell lymphoma (SCL). The etiopathogenesis of these diseases is not fully understood; however, it is hypothesised that it involves a combination of factors such as genetic susceptibility, environmental triggers, altered immune regulation and altered composition and/or functionality of the intestinal microbiome [2].

Microorganisms inhabiting the gastrointestinal tract (GI) form a complex ecosystem that influences the physiology of the host. Among all microorganisms, bacteria make up the majority of the gastrointestinal microbiota and account for over 98% of metagenomics sequencing reads in faecal samples obtained from dogs and cats [3, 4], with Firmicutes and Bacteroidota being usually the predominant phyla, followed by Actinobacteria in cats [5–7]. Although most mammals share similar bacteria at the phylum level [8], assessing the microbes at lower taxonomic levels may play an important role in understanding the inter- and intraspecific differences. Novel molecular methods allow more in-depth insight into the GI microbiota, with sequencing of 16 S RNA gene being the most commonly used method for microbiota assessments [9]. Alterations in the gut microbiome (both the composition of the microbiota and its functionality) are observed following environmental changes (diet) and when considering individual factors (age), but most prominent alterations are found in diseased animals, especially those suffering from GI disease [10].

The coevolution of gastrointestinal microbiota and mammals has resulted in a variety of types of interactions, from digestion of fibre and providing nutrients to enterocytes to a pivotal role in regulating immune responses in the host [11]. Therefore, studies on the microbiome are based not only on microbiota assessments but also on the evaluation of bacterial metabolites as factors modulating immune response. One well-known example of this relationship is the fermentation of dietary carbohydrates into short-chain fatty acids (SCFAs), which become not only an energy source for the epithelial cells of the gastrointestinal tract, but also influence smooth muscle contraction [12] and take part in immunomodulation [13]. These effects are mediated mainly by the major SCFAs: acetic acid, propionic acid and butyric acid whereas branched-chain fatty acids (BCFAs), e.g., isovaleric and isobutyric acid, produced during the process of protein fermentation in the colon are believed to promote intestinal inflammation [14]. Observations of changes in SCFAs proportion or concentration have come mainly from studies of human patients [15, 16], and the mechanisms of their interactions with the immune system are studied mainly on laboratory animal models [17–19]. To date, only a few studies have addressed this issue in dogs and cats although the GI tract of carnivores (especially obligatory, such as domestic cats) vary widely from the GI tract of omnivores, with a relatively short GI tract and lower dependence on microbial fermentation as an energy source [20]. Therefore, studies on other species, including dogs and cats, are needed to provide more insight into species-specific characterization of the GI microbiome.

## Results

### Alpha diversity measures

**Fig. 1:**
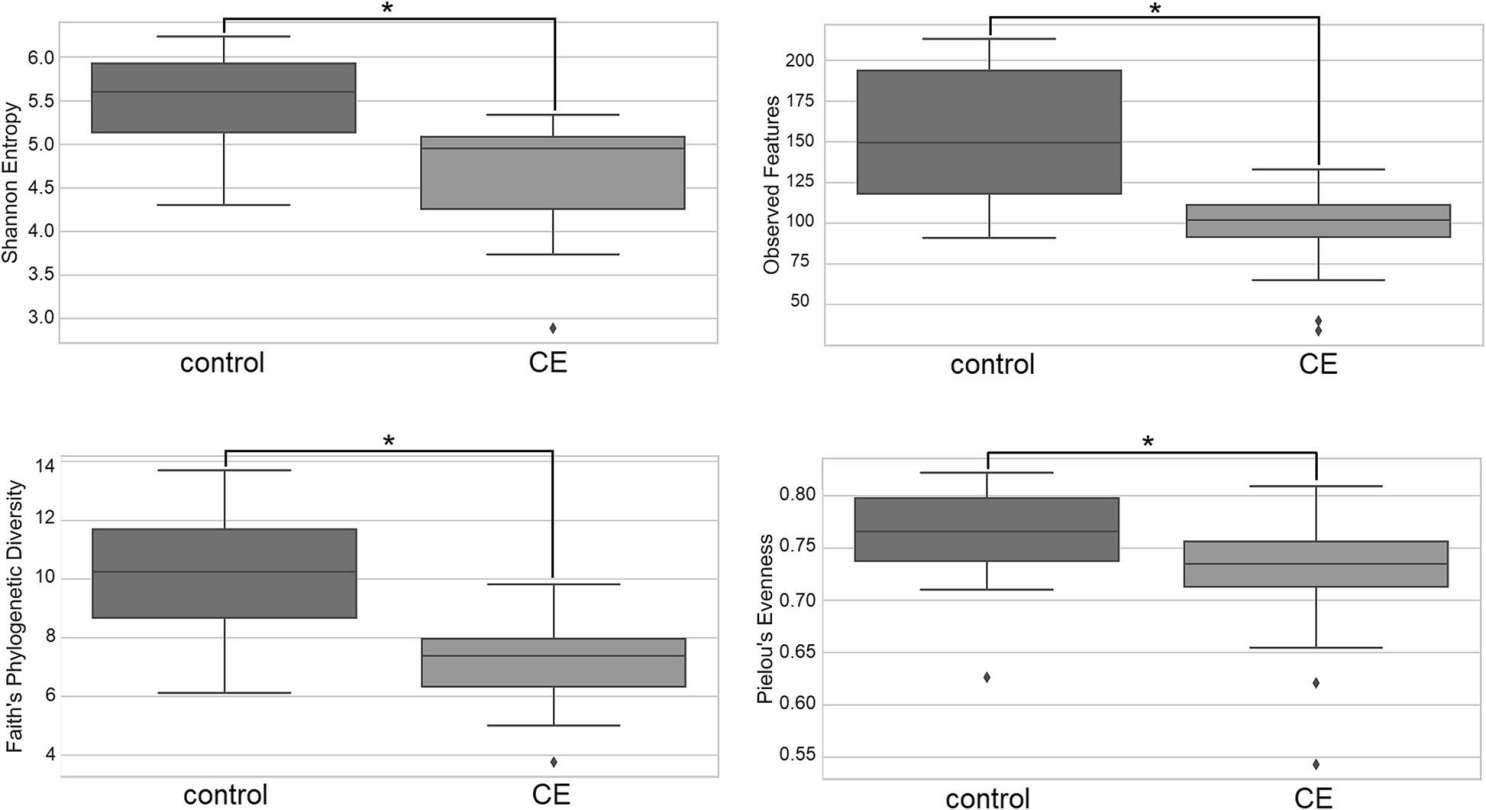
Alpha diversity measures evaluating bacterial distributions in stool samples from control cats and cats with chronic enteropathy (CE). The box shows the quartiles of the dataset. Bottom whisker is the minimum, upper whisker is the maximum. The outliers are marked with rhombus. Statistical differences were observed in all four metrics and are marked with asterisks

Statistically significant differences in the alpha diversities were observed using Kruskal-Wallis test in the microbiota of the stool samples of control cats (*n* = 14) and cats with CE (*n* = 16), as measured with the Shannon’s diversity index: mean 5.50 (± 0.58) vs. 4.70 (± 0.67), *p* < 0.01; observed features: 155.57 (± 40.53) vs. 95.25 (± 27.71), *p* < 0.01; and Faith’s phylogenetic diversity: 10.06 (± 2.15) vs. 7.17 (± 1.60), *p* < 0.01, control vs. CE, respectively. There were also significant differences in the bacterial evenness between these two groups; in the stool samples of control cats, the bacteria were slightly more evenly distributed, with Pielou’s evenness: 0.76 (± 0.05) vs. 0.72 (± 0.07), *p* < 0.05. (Fig. 1). The raw data are available in Supplemental Table 1 (S1).

### Beta diversity measures

**Fig. 2:**
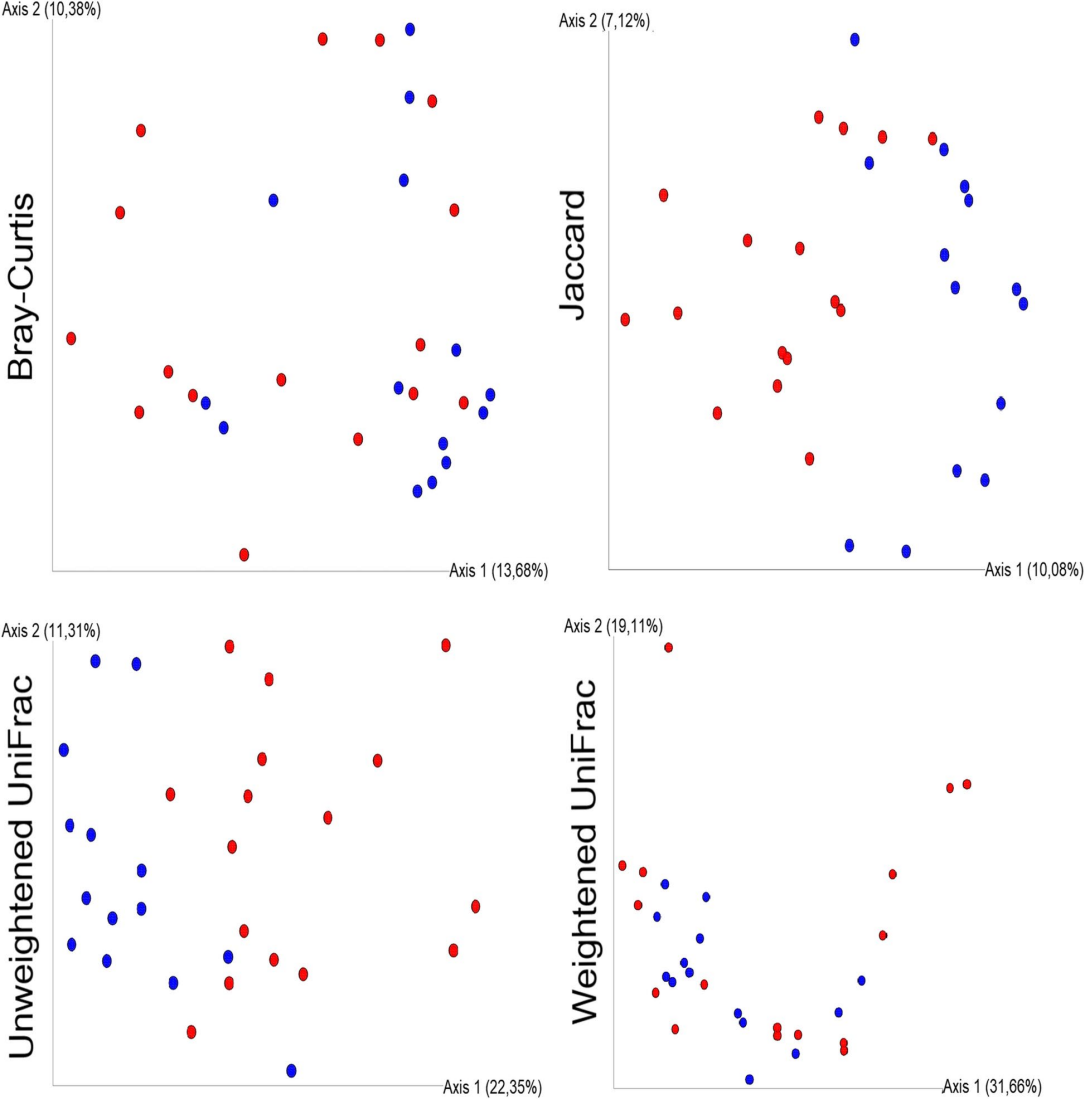
Beta diversity. Principal coordinate analysis (PCoA) plot showing clustering of microbial communities from the faeces of control and chronic enteropathy (CE) cats (red = CE, blue = control). Statistical differences were noted in all four metrics evaluated

There were significant changes in the ecological distances among the microbiota in the faecal samples obtained from the control cats and the microbiota in the faecal samples obtained from cats with CE for all four metrics evaluated: Bray‒Curtis, *p* < 0.02; Jaccard, *p* < 0.01; unweighted UniFrac, *p* < 0.01; and weighted UniFrac, *p* < 0.05; PERMANOVA test, 999 permutations (Fig. 2).

The ANCOM method revealed differences in the microbiota compositions between control cats and cats with CE.

At the phylum level, Proteobacteria was more abundant in cats with CE (W = 1), and Bacteroidota was more abundant in control cats (W = 1).

No significant changes were detected at the class level.

At the order level, Enterobacterales was more abundant in cats with CE (W = 31), as well as Lactobacillales (W = 26). The order Peptococcales was more abundant in the faeces of control cats (W = 25).

At the family level, *Enterobacteriaceae* was more abundant in the faeces of cats with CE than in the faeces of control cats, where it was barely detected (W = 52), and at the genus level, *Escherichia Shigella* was more abundant in samples from cats with CE (W = 176).

**Fig. 3:**
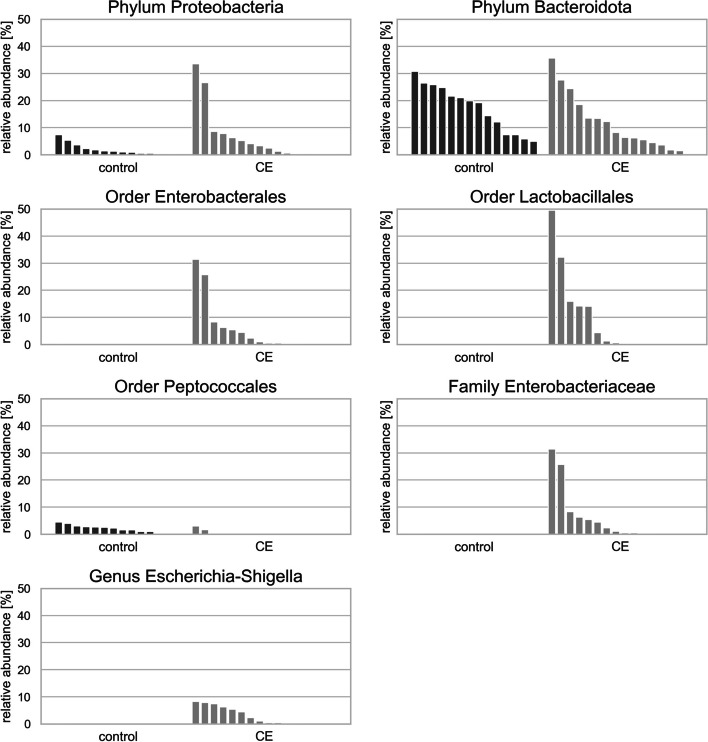
Statistically significant differences in the compositions of faecal microbiota at the phylum, order, family and genus levels. *CE* Chronic enteropathy

The significantly differentially abundant taxa are shown in Fig. 3.

### Fatty acids

The SCFA concentrations (µmol / 1 g ) were significantly higher in samples obtained from cats with CE (median 36.08) than in samples obtained from control cats (median 11.45), *U* = 33, *p* < 0.01.

**Fig. 4:**
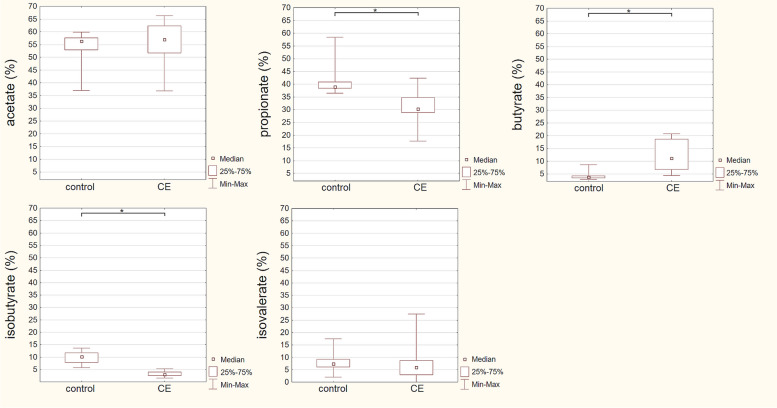
Median proportions of faecal acetate, propionate butyrate, isobutyrate and isovalerate in control cats and cats suffering from chronic enteropathy (CE). Statistically significant differences are marked with an asterisk

The propionate proportion was significantly higher in the control cats vs. CE cats: median 38,99% vs. 30,28%, *U* = 18, *p* < 0.01, while the butyrate proportion was statistically lower in the control cats vs. CE cats: median 3.08% vs. 10.21%, *U* = 7, *p* < 0.01. The acetate proportion did not differ significantly (*p* > 0.05) between groups with a median of 45.86% in the control cats and 53.25% in cats with CE (Fig. 4).

Statistically significant differences were also observed for the proportion of isobutyric acid, with a higher proportion in control cats: median 10.18% vs. 2.96% in the control and CE cats, respectively, U = 0, *p* < 0.01. No significant differences (*p* > 0.05) were observed in the isovalerate proportions: median 7.37% in the control group vs. 6.03% in CE cats (Fig. 4).

**Fig. 5:**
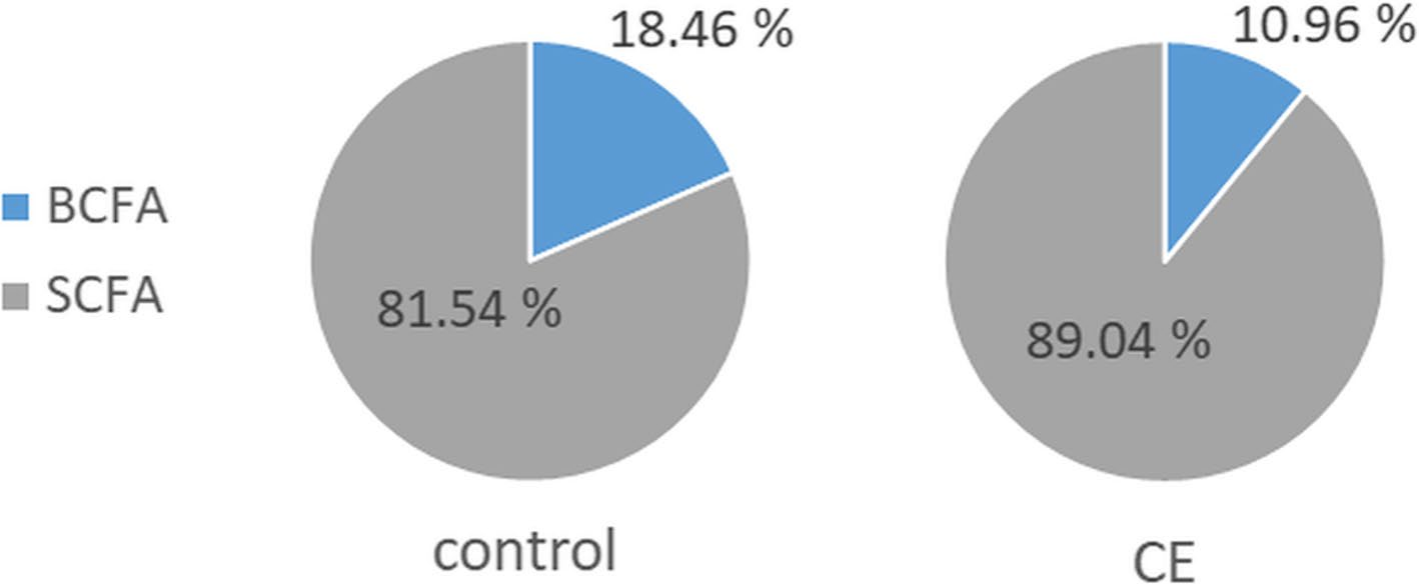
Mean proportions of faecal BCFAs and SCFAs in control cats and cats suffering from chronic enteropathy (CE). The differences were statistically significant

 The SCFA/BCFA ratios were significantly lower in the control cats vs. CE cats: 5.91 (± 2.27) vs. 13.67 (± 6.34), *p* < 0.01. When expressed as the percentages of total fatty acids, the proportions of BCFAs were 18.46% (± 4.75) in the control cats and 10.96% (± 7.80) in CE cats and differed significantly between the groups (*p* < 0,05) (Fig. 5).

Raw data showing the fatty acid concentrations are available as a supplemental file (S2).

## Discussion

Our study revealed lower diversity of the faecal microbiota in cats suffering from CE, which is a common finding in studies evaluating faecal microbiota in GI disorders in other species, such as dogs [21, 22] and humans [23, 24]. However, similar studies have shown inconsistent results. Suchodolski and colleagues found no statistical difference in the species richness and microbial diversity in faecal samples obtained from dogs suffering from both acute and chronic GI disease when compared to healthy dogs, although a trend toward lower species richness and microbial diversity was observed in diseased animals [25]. A study conducted on a larger group of lymphoma and IBD cats found no significant differences in the microbiota (both diversity and composition) between those two groups, but found significant differences when samples from diseased animals were compared with those obtained from healthy animals [26]. Due to a lack of final histopathological diagnoses in all cases and relatively small sample sizes, we were not able to provide a reliable comparison of lymphoma and IBD cases. However, there is an ongoing debate regarding whether IBD and lymphoma are two distinct diseases or represent different points of a disease spectrum because of the well-known hypothesis that IBD can progress to lymphoma in some cats [2] In another study in which cats were enrolled based on clinical signs, cats with diarrhoea also showed lower alpha diversity scores, regardless of whether the diarrhoea was classified as acute or chronic [27]. In our study, the diversity was significantly lower in cats with CE, although diarrhoea was only one of the possible clinical signs considered during patient enrolment.

A higher relative abundance of Proteobacteria (or particularly Enterobacteriaceae) is another observation that is consistent with the results obtained in other studies on the gut microbiome in human [28, 29], feline [26, 27, 30] and canine enteropathies [21, 31, 32]. In human medicine, members of the phylum Proteobacteria are considered to be a “microbial signature of dysbiosis” [33]. At the genera level, *Escherichia Shigella* was found to be enriched in human patients with Crohn’s disease [29]. This observation is consistent with our findings; however, in the cited study, the authors examined both stool and mucosal samples. Similarly, a study on the spatial distribution of colonic bacteria in dogs suffering from chronic inflammatory enteropathy showed higher *Escherichia Shigella* abundance both on the colonic surface and within the crypts in samples obtained from diseased dogs [34].

Lower Bacteroidota abundance in animals suffering from GI disease is also a phenomenon previously described both in cats [26, 27] and dogs [22, 35]. Similar results were shown in a study using fluorescence in situ hybridization in faecal samples from cats with IBD [36]. While this result is consistent with our findings, other differences, for example, lower *Bifidobacterium* spp. counts and higher *Desulfovibrio* spp. counts, were not confirmed in our research.

Although we did not observe differences in Firmicutes relative abundance at the phylum level, the CE samples showed a higher relative abundance of Lactobacillales and a lower relative abundance of Peptococcales. In a study assessing the abundance of bacteria along the feline intestine, Lactobacillales were found mainly in the jejunum [37], which is an interesting finding, as higher abundance of these bacteria in the faeces of diseased cats in our study could reflect potential dysbiosis in the small intestine. This hypothesis could not be confirmed in our study, as we did not obtain samples from different compartments of the intestine. What is more, a study using group-specific primers for *Bifidobacterium* and *Lactobacillus* detected *Lactobacillus* spp. in 92% of cats vs. 13.3% of positive samples when using universal bacterial primers [38]. This observation clearly shows that comparisons of results obtained from different studies can easily be misinterpreted due to potential differences in methodologies. To our knowledge, there is no data on the role of Peptococcales in the intestinal microbiome of cats. Studies in other species, as well as in vitro studies, show that members of this family are considered sulphate-reducing bacteria or mucin degraders [39, 40]. These two features should have a detrimental effect on the integrity of mucosal membranes, and there are studies confirming the link between sulphate-reducing bacteria and intestinal inflammation; however, the bacteria identified were mostly from other orders [41]. What is more the role of sulphate-producing bacteria can be different in species that are obligate carnivores consuming a high-protein diet.

Changes in the microbiota are important because of their interplay with the immune system, with SCFAs production being one of the important factors influencing the host. The higher SCFAs concentration in CE cats was an unexpected finding, as studies conducted mainly on other species show the opposite effect when comparing healthy patients with patients suffering from GI disease. Examples include food-responsive and chronic enteropathy in dogs as well as IBD in humans [22, 42, 43]. Comparisons of SCFAs among species have to be done with caution as in human patients with IBD typically the large bowel is affected, whereas in cats the small intestine is more commonly affected. Thus, colonic SCFA production in feline patients might be less affected by IBD, whereas the faecal SCFA concentration might be higher due to increased elimination in animals suffering from diarrhoea. Another possible explanation is altered substrate availability for colonic fermentation in patients suffering from digestion disturbance in the small intestine.

An important issue to address is also the impact of sampling procedures and sample handling. In our study, samples were mainly collected by animal owners after natural voiding of faeces, and although they received uniform instructions, differences in sample handling cannot be ruled out. Therefore, to ensure maximum reliability of the results, particular SCFAs were expressed not in absolute concentration, but in ratios. This decision was based on a methodological study by Cunningham and collegues [44], which revealed that single SCFA concentrations were highly impacted by temperature and time after sampling, whereas both the SCFAs ratios and microbiota composition were much more stable. Regarding the presentation of the results of SCFAs quantities, in the majority of studies performed on human and laboratory models the concentration is expressed as mmol/l or µmol/g faeces, but another possible method involves adjusting for the dry faecal mass. As presented by Minamoto and colleagues in their study on dogs with CE, the results of the statistical analysis of SCFAs (mainly butyrate) concentration differed depending on which value was assessed [22].

Among the SCFAs, propionate and butyrate are considered to be the most important for gastrointestinal health and epithelial cell nutrition. Propionate is an important anti-inflammatory mediator, which decreases the production of proinflammatory cytokines [19] and regulates the homeostasis of colonic T regulatory cells [13]. In our study, a reduced proportion of propionate in cats with CE was accompanied by a lower relative abundance of Bacteroidota, which are important propionate producers [45]. Similar to our results, a reduced proportion of propionate was also observed in dogs with CE [22]. Although butyrate is considered another SCFA that is important for maintaining gut health and exhibits anti-inflammatory properties [46], in our study, the proportion of butyrate in faecal samples was even higher in the CE group than in healthy cats. Interestingly, Minamoto et al. [22] found no significant differences in butyrate concentration in the dry mass of faeces, despite significantly lower abundance of potential butyrate-producing bacteria in dogs with CE. Butyrate levels were higher in faecal samples obtained from diseased animals in a study on the microbiome and metabolome of dogs with acute diarrhoea [47]. This was also an unexpected finding, as the authors found a lower abundance of classic butyrate producers in dogs with acute diarrhoea. The authors suggested a possible decrease in butyrate absorption or utilization by enterocytes. This may also partly explain our results, however, diarrhoea is not the predominant clinical symptom of feline CE as vomiting and inappetence are more common. Other possible explanations are different patterns of SCFA production and utilization by colonic cells in obligate carnivores or a distinct mechanism of butyrate production in animals suffering from diseases of the small intestine.

Branched chain fatty acids are less frequently assessed in faecal samples in GI diseases. Their production in the human intestine is carried out mainly by the genera *Bacteroides* and *Clostridium* [48]. Moreover, higher concentrations of BCFAs were found in faecal samples of human patients receiving high-protein diet [14]. In our study we were not able to evaluate the protein content of the cats’ diet, however, it is obvious that the feline diet is rich in proteins and therefore it is an important factor influencing BCFA production.

The limitations of our study include the relatively low number of samples and no histopathological diagnosis in four cases. Another problem was the sampling procedure, which was performed by the cat owners and could not be controlled directly by a member of the research group. Additionally, six animals were receiving oral medication at the time of sampling. We decided not to exclude those animals, as according to other studies prednisolone does not significantly affect the faecal microbiome [49], and the effects of metronidazole, even though they are known to have a greater impact on the intestinal microbiome, are usually studied in 14-day trials [49, 50] Additionally, in a study on faecal SCFAs in dogs with CE, an analysis of the medical treatment history revealed no significant changes in the concentration of SCFAs between dogs receiving no treatment and those receiving antibiotics, immunosuppressive agents or both [22]. For this reason we have included patients that received metronidazole only recently (up to five days prior to sampling). Nevertheless, the medical treatment history is an important factor to consider and is a major problem in studies based on clinical cases.

## Conclusions

The results of our study revealed alterations in microbiota composition and fatty acid production in cats suffering from CE. This is important in the context of the growing interest in novel treatment options, including faecal microbiota transplantation, probiotic or dietary supplementation and avoidance of antibiotics and immunosuppressants overuse. The limited number of studies on the microbiome of diseased cats and usually a relatively small number of samples makes it complex to discuss the results, but these difficulties point to the need for more intensive research in this area, as well as to standardize the data presentation.

## Methods

### Animals

Thirty privately owned cats were enrolled in the study.

The control group consisted of 14 cats (nine males, five females, age 8.6 ± 1.6) that were deemed healthy based on anamnesis (no signs of gastrointestinal problems in the last six months) and physical examinations, basic biochemistry profiles, complete blood counts, faecal examinations by flotation tests, rapid enzyme immunochromatographic Giardia assays and abdominal ultrasounds.

Sixteen cats (ten males, six females, age 9.6 ± 4.0 years) suffering from clinical symptoms of CE were included in the study. The basic diagnostic evaluation included clinical signs of enteropathy for at least three weeks (e.g., vomiting, diarrhoea, anorexia, and weight loss), basic biochemistry profiles, complete blood counts, faecal flotation tests, rapid enzyme immunochromatographic Giardia assays and abdominal ultrasound examinations. Histopathologic findings from samples obtained by laparotomy were available for four animals at the time of sample collection (animals that were presented for control visits), and eight additional histopathological findings were included after obtaining a final diagnosis.

At the time of sample collection, three animals were receiving long-term immunosuppressive therapy (prednisolone), and three animals were receiving metronidazole (up to five days prior to sampling). Patients receiving antibiotics for longer than five days, patients receiving probiotics as well as animals that were under owners care for less than 6 months were excluded from the study. All animals were fed commercial diet.

Based on the histopathological findings, the study group included six cats with alimentary tract SCL, six cats with IBD and four cats with unidentified chronic enteropathy (no histopathological examinations were performed).

In two cases (i.e., one control cat and one cat with CE), the amount of faeces collected by the owners was relatively small and for these samples, only microbiota analyses were performed (for fatty acid analyses, the sample numbers were 13 in the control group and 15 in the CE group).

### Sampling procedure

Samples were collected up to six hours after natural voiding of faeces. The samples were stored in a refrigerator for a maximum of 12 h after collection, shipped to the laboratory on ice and frozen in 1-g portions at -80 °C prior to analysis. If a sample could not be shipped within 12 h, it was frozen immediately at -20 °C after sampling and shipped on ice to the laboratory. Cryogenic storage tubes were used to store all samples.

### Analysis of SCFAs

Analyses of derivatized stool extracts by high-performance liquid chromatography (HPLC) were performed according to Torii et al. [51]. Faecal samples were extracted with 70% ethanol. After centrifugation, debris was removed, and 500 µL of supernatant was transferred to a new tube. Afterwards, the supernatant was mixed with 50 µL of internal standard (2-ethylbutyric acid, 200 mM in 50% aqueous methanol), 300 µL of dehydrated pyridine 3% v/v (Merck, Darmstadt, Germany) in ethanol, 300 µL of 250 mM N-(3-dimethlaminopropyl)-N′;-ethylcarbodiimide hydrochloride (Merck) in ethanol, and 300 µL of 20 mM 2-nitrophenylhydrazine hydrochloride (Merck) in ethanol. After the first incubation (60 °C, 20 min), 200 µL of potassium hydroxide solution (15% w/v with water and with a potassium hydroxide solution/methanol ratio of 80/20) was added and incubated at 60 °C for 20 min. In the next step (after cooling), the samples were shaken with three ml of phosphoric acid aqueous solution (0.5 mol/L) and four ml of ether two times for three minutes. The organic phase was extracted by shaking with four ml of diethyl ether and then transferred to a new glass conical vessel containing water to extract any remaining aqueous compounds. The fatty acid hydrazides were dissolved in 100 µL of methanol, and finally, 20 µL was subjected to HPLC [51, 52].

HPLC was performed using a 1525 Binary HPLC Pump with a 2489 UV/Visible (UV/Vis) Detector and Phenomenex Gemini® 5 μm C18 110 Å (150 × 4.6 mm) (Phenomenex, Aschaffenburg, Germany). The mobile phase was composed of acetonitrile-methanol-water (30:16:54), and the pH was adjusted to 4.5 with 0.1% trifluoroacetic acid. The column temperature was 50 °C with a flow rate of one ml/min and a measurement wavelength of 400 nm was used [51, 52]. The following fatty acids were measured: acetic acid, propionic acid, butyric acid, isobutyric acid and isovaleric acid. The results of the overall concentration of SCFAs are expressed as µmol/1 g of faeces.

The proportions of the major SCFAs were expressed as follows:

$$\%\;\mathrm{acetate}\;=\;\mathrm{acetate}\;\mathrm{concentration}/\mathrm{acetate}\;+\;\mathrm{propionate}\;+\;\mathrm{butyrate}\;\mathrm x\;100\%$$

$$\%\;\mathrm{propionate}\;=\;\mathrm{propionate}\;\mathrm{concentration}/\mathrm{acetate}\;+\;\mathrm{propionate}\;+\;\mathrm{butyrate}\;\mathrm x\;100\%$$

$$\%\;\mathrm{butyrate}\;=\;\mathrm{butyrate}\;\mathrm{concentration}/\mathrm{acetate}\;+\;\mathrm{propionate}\;+\;\mathrm{butyrate}\;\mathrm x\;100\%$$

The proportion of BCFAs (isobutyric + isovaleric acid) was determined by using the sum of the concentrations of all SCFAs evaluated.

The proportions were determined in two different ways because most studies concentrate on the major SCFAs produced in the gut (e.g., acetate, propionate, and butyrate), and these values are easier to compare. The proportions of acetate, propionate and butyrate that were used to calculate the sum of all SCFAs are available in the Supplementary information.

The BCFA/SCFA ratio was determined as follows: isobutyric + isovaleric acid/acetate + propionate + butyrate + isobutyric + isovaleric.

Statistical analyses of the results were conducted using Statistica 13 (StatSoft Tulsa, USA).

The data were checked for normality using the Lilliefors test. Values with normal distributions were analysed using Student’s t-tests and presented as mean ± standard deviation. Mann Whitney *U* test was used for values without normal distributions and these results were presented as medians. The statistical results were considered significant when the p value was below 0.05.

### Analysis of microbiota

To extract microbial DNA from the frozen stool samples, a QIAamp PowerFecal Pro DNA kit (Qiagen, Hilden, Germany) was used. To prepare the DNA for next-generation sequencing, the QIAseq 16 S/ITS Region Panel for the hypervariable region V3V4 (Qiagen) was used according to the manufacturer’s protocol. Paired-end sequencing of the 16 S rRNA gene amplicon libraries was performed using a MiSeq Reagent Kit v3 for 600 cycles (Illumina, San Diego USA), and FASTQ files were obtained.

For the microbiota analyses, the bioinformatics platform, QIIME2 (2021.8), with supplementary plugins was used [53]. To cut the V3V4 primers and perform demultiplexing, a custom script that used *cutadapt* was applied [54]. The *summary* method in the *demux* plugin was used to evaluate the quality of the reads. Trimming, denoising, dereplication, and chimaera filtering were performed with the *dada2* plugin for paired-end reads [55]. To construct the phylogenetic tree, the *q2-phylogeny align-to-tree-mafft-fasttree* single routine was used. This procedure uses the *mafft* method to align multiple sequences, masks highly variable positions, and *fasttree* then constructs a phylogenetic tree [56, 57]. Rarefaction (subsampling without replacement) was performed on up to 21,218 sequences per sample. The *q2-diversity* plugin was used to estimate the alpha (α) and beta (β) diversities and to perform principal coordinate analysis (PCoA) (α-diversity metrics: Shannon’s diversity index, observed features, Faith’s phylogenetic diversity, and Pielou’s evenness; β-diversity metrics: Jaccard similarity index, Bray-Curtis dissimilarity, unweighted UniFrac and weighted UniFrac) [58–60]. To assign taxonomy to the amplicon sequence variants (ASVs), the naive Bayesian classifier was trained on fragments of the 16 S rRNA gene sequences derived from the SILVA 138 SSURef NR99 database with the use of the method *fit-classifier-naïve-Bayes* from the f*eature-classifier* plugin [61–64]. To identify the differentially abundant features among groups, the ANCOM (analysis of the composition of microbiomes) tool was used as implemented in the q2-composition plugin with default parameters [65].

The descriptive statistics are presented as the means and standard deviations. The parameter differences between groups were analysed using the Kruskal‒Wallis test with Holm correction post hoc analysis. To test the beta-diversity group significance, PERMANOVA with 999 permutations was conducted. All calculations were conducted using the R package for Windows (version 4.2, R Core Team). The statistical results were considered significant if the p-values were less than 0.05.

### Supplementary Information

**Additional file 1: Supplementary Table 1.** Alpha diversity measures in individual samples

** Additional file 2: Supplementary Table 2.** Fatty acids concentrations in individual samples

## Data Availability

Raw data are available as supplementary material. Other datasets used during the current study (ex. full set of graphs from ANCOM analysis) are available from the corresponding author on reasonable request.
