# Supplementary material for: Faecal microbiota and fatty acids in feline chronic enteropathy
Source: BMC Vet Res. 2023 Dec 20;19:281. doi: 10.1186/s12917-023-03824-9 (PMC10731866; doi:10.1186/s12917-023-03824-9)
Supplement: Supplementary file 1 — Additional file 1: Supplementary Table 1. Alpha diversity measures in individual samples [file 12917_2023_3824_MOESM1_ESM.docx]

| name | group | sample-id | shannon_entropy | observed_features | pielou_evenness | faith_pd |
| --- | --- | --- | --- | --- | --- | --- |
| CC 1 | control | 1 | 5.422176 | 142 | 0.7583731 | 10.603285 |
| CC 2 | control | 2 | 5.2327513 | 116 | 0.7630163 | 7.2112316 |
| CC 3 | control | 3 | 4.7816508 | 89 | 0.7383952 | 6.0322495 |
| CC 4 | control | 4 | 5.8579719 | 191 | 0.7730817 | 10.239032 |
| CC 5 | control | 5 | 6.0120589 | 175 | 0.8068566 | 11.231106 |
| CC 6 | control | 6 | 4.8744178 | 118 | 0.7082189 | 8.8059526 |
| CC 7 | control | 7 | 5.862876 | 153 | 0.8078493 | 9.2470343 |
| CC 8 | control | 8 | 5.9428867 | 212 | 0.769015 | 11.801441 |
| CC 9 | control | 9 | 4.2999341 | 117 | 0.6258669 | 8.6359223 |
| CC 10 | control | 10 | 6.2095257 | 192 | 0.8186627 | 12.340258 |
| CC 11 | control | 11 | 5.5355133 | 146 | 0.7699094 | 10.252401 |
| CC 12 | control | 12 | 5.1132713 | 119 | 0.7416108 | 8.3419148 |
| CC 13 | control | 13 | 5.6560175 | 205 | 0.7365105 | 13.69813 |
| CC 14 | control | 14 | 6.1993086 | 203 | 0.8087459 | 12.34756 |
| **Mean:** | | | **5.500026** | **155.5714** | **0.759008** | **10.05625** |
| *Std:* | | | *0.575848* | *40.52865* | *0.050139* | *2.148134* |

| name | group | sample-id | shannon_entropy | observed_features | pielou_evenness | faith_pd | |
| --- | --- | --- | --- | --- | --- | --- | --- |
| CE 1 | CE | 15 | 5.0770353 | 114 | 0.743029 | 9.7600205 | |
| CE 7 | CE | 21 | 2.8871613 | 40 | 0.5425029 | 5.0045383 | |
| CE 8 | CE | 22 | 4.1925152 | 108 | 0.620664 | 8.689619 | |
| CE 10 | CE | 24 | 5.3391357 | 97 | 0.8089706 | 7.1088809 | |
| CE 15 | CE | 29 | 3.7421774 | 34 | 0.7355685 | 3.7622915 | |
| CE 16 | CE | 30 | 5.0699918 | 110 | 0.7476365 | 7.7519914 | |
| CE 2 | CE | 16 | 5.0989046 | 124 | 0.7332126 | 7.7266611 | |
| CE 3 | CE | 17 | 5.2496745 | 115 | 0.7668807 | 8.1336848 | |
| CE 5 | CE | 19 | 5.0885785 | 134 | 0.7201407 | 9.3792853 | |
| CE 9 | CE | 23 | 4.7988164 | 92 | 0.7356129 | 7.1156848 | |
| CE 11 | CE | 25 | 4.2507036 | 90 | 0.6547751 | 7.5290379 | |
| CE 12 | CE | 26 | 4.2626093 | 65 | 0.7077962 | 5.1477851 | |
| CE 13 | CE | 27 | 5.3495298 | 108 | 0.7919495 | 7.2439872 | |
| CE 14 | CE | 28 | 4.9727121 | 93 | 0.7604513 | 7.8701899 | |
| CE 4 | CE | 18 | 4.8035039 | 107 | 0.7125309 | 6.0207292 | |
| CE 6 | CE | 20 | 4.9445297 | 93 | 0.7561416 | 6.4325994 | |
| **Mean:** | | | **4.695474** | **95.25** | **0.721116** | **7.167312** |  |
| *Std:* | | | *0.669853* | *27.71402* | *0.066332* | *1.599219* |  |

Supplementary Table 1. Alpha diversity measures in individual samples

Raw data showing the results of alpha-diversity measures in faecal samples obtained from healthy cats (CC) and cats suffering from chronic enteropathy (CE ).
