# Supplementary material for: Faecal microbiota and fatty acids in feline chronic enteropathy
Source: BMC Vet Res. 2023 Dec 20;19:281. doi: 10.1186/s12917-023-03824-9 (PMC10731866; doi:10.1186/s12917-023-03824-9)
Supplement: Supplementary file 2 — Additional file 2: Supplementary Table 2. Fatty acids concentrations in individual samples [file 12917_2023_3824_MOESM2_ESM.docx]

|  | **acetate µmol / 1g** | **propionate µmol / 1g** | **butyrate µmol / 1g** | **isobutyrate µmol / 1g** | **isovalerate µmol / 1g** |
| --- | --- | --- | --- | --- | --- |
| **CE 1** | 5.916 | 6.806 | 3.325 | 1.189 | 6.543 |
| **CE 2** | 36.755 | 22.64 | 13.669 | 2.832 | 5.841 |
| **CE 3** | 19.757 | 10.06 | 3.41 | 0.921 | 1.571 |
| **CE 4** | 19.212 | 10.973 | 3.498 | 1.166 | 1.231 |
| **CE 5** | 38.401 | 16.274 | 6.869 | 1.69 | 0 |
| **CE 6** | 57.593 | 33.06 | 20.745 | 3.088 | 3.541 |
| **CE 8** | 3.474 | 1.91 | 0.699 | 0.194 | 0.567 |
| **CE 9** | 29.238 | 7.777 | 7.032 | 1.451 | 3.424 |
| **CE 10** | 5.325 | 2.801 | 1.996 | 0.693 | 2.229 |
| **CE 11** | 16.13 | 8.37 | 4.443 | 0.846 | 4.592 |
| **CE 12** | 30.8 | 17.648 | 2.319 | 0.836 | 1.211 |
| **CE 13** | 110.571 | 71.389 | 12.335 | 3.711 | 4.828 |
| **CE 14** | 1.921 | 0.856 | 0.128 | 0.099 | 0.289 |
| **CE 15** | 19.122 | 14.751 | 3.792 | 1.633 | 2.081 |
| **CE 16** | 13.502 | 6.294 | 1.43 | 1.246 | 1.441 |
|  |  |  |  |  |  |
| **CC 1** | 10.401 | 9.59 | 0.765 | 2.522 | 1.52 |
| **CC 3** | 10.248 | 16.162 | 1.244 | 1.736 | 0.596 |
| **CC 4** | 3.262 | 2.161 | 0.233 | 0.469 | 0.487 |
| **CC 5** | 2.767 | 2.007 | 0.454 | 0.735 | 1.257 |
| **CC 6** | 4.993 | 4.378 | 0.419 | 0.927 | 1.411 |
| **CC 7** | 8.552 | 5.703 | 0.524 | 1.674 | 0.976 |
| **CC 8** | 6.741 | 4.608 | 0.499 | 1.682 | 1.168 |
| **CC 9** | 5.254 | 3.742 | 0.315 | 1.173 | 0.964 |
| **CC 10** | 5.315 | 3.897 | 0.323 | 1.414 | 1.1 |
| **CC 11** | 4.206 | 2.559 | 0.254 | 1.181 | 0.486 |
| **CC 12** | 3.702 | 2.581 | 0.336 | 0.685 | 1.439 |
| **CC 13** | 4.679 | 2.901 | 0.223 | 1.134 | 0.653 |
| **CC 14** | 3.293 | 2.35 | 0.194 | 0.854 | 0.453 |

Supplementary Table 2. Fatty acids concentrations in individual samples

Raw data showing the concentration of fatty acids in faecal samples obtained from healthy cats (CC) and cats suffering from chronic enteropathy (CE).
